# Conformational Dynamics and Structural Transitions of Arginine Kinase: Implications for Catalysis and Allergen Control

**DOI:** 10.3390/life15081248

**Published:** 2025-08-06

**Authors:** Sung-Min Kang

**Affiliations:** College of Pharmacy, Duksung Women’s University, Seoul 01369, Republic of Korea; smkang@duksung.ac.kr

**Keywords:** arginine kinase, structural transition, transition state analog, allergen control

## Abstract

Arginine kinase is a key phosphagen kinase in invertebrates that facilitates rapid ATP regeneration by reversibly transferring phosphate groups between phosphoarginine and ADP. Structural studies have shown that the enzyme adopts distinct conformations in its ligand-free and ligand-bound states, known as the “open” and “closed” forms, respectively. These conformational changes are crucial for catalytic activity, enabling precise positioning of active-site residues and loop closure during phosphoryl transfer. Transition-state analog complexes have provided additional insights by mimicking intermediate states of catalysis, supporting the functional relevance of the open/closed structural model. Furthermore, studies across multiple species reveal how monomeric and dimeric forms of arginine kinase contribute to its allosteric regulation and substrate specificity. Beyond its metabolic role, arginine kinase is also recognized as a major allergen in crustaceans. Its structural uniqueness and absence in vertebrates make it a promising candidate for selective drug targeting. By integrating crystallographic data with functional context, this review highlights conserved features and species-specific variations of arginine kinase that may inform the design of inhibitors. Such molecules have the potential to serve both as antiparasitic agents and as novel therapeutics to manage crustacean-related allergic responses in humans.

## 1. Introduction

To date, dozens of arginine kinase structures have been deposited in the Protein Data Bank (PDB), and several of the earlier entries were originally annotated under the name creatine kinase [1]. These structures exhibit high structural similarity, reflecting their strong sequence conservation [2]. Each monomer typically consists of a small N-terminal domain comprising approximately 100 residues and a larger C-terminal domain of around 280 residues. The ATP-binding site is located within the cleft formed between these two domains [3].

Phosphagen kinases, including arginine kinase and creatine kinase, utilize phosphoarginine and phosphocreatine, respectively, as phosphagen compounds to rapidly supply energy in animals [4]. Phosphagen kinases are broadly distributed across the animal kingdom and have also been identified in some unicellular organisms, protists, and bacteria, although they are most commonly found in multicellular animals [5]. In certain physiological conditions where immediate energy production is required, phosphagens support cellular activity until catabolic pathways such as glycolysis or oxidative phosphorylation are activated to restore ATP levels [6]. Phosphagens like phosphoarginine, phosphocreatine and phosphotaurocyamine function as energy reservoirs, quickly donating their high-energy phosphate groups to ADP to regenerate ATP in response to sudden energy demands [7].

Arginine kinase, a homolog of creatine kinase, is predominantly found in arthropods and other invertebrates [6]. Arginine kinase is primarily found in muscle and heart tissues, but it is also expressed in gills, intestine, digestive glands and eggs [8,9]. Arginine kinase catalyzes the reversible phosphorylation of arginine within the cell and functions either as a monomeric or dimeric enzyme [10]. Structural analyses have revealed significant positional shifts in certain residues upon substrate binding, often involving a flexible loop that moves to cover the ligand-binding pocket [11]. Along with creatine kinase, it also plays an important role in maintaining energy homeostasis by transporting energy between different cellular compartments [12].

Creatine kinase is an enzyme of the phosphagen kinase family that catalyzes the reversible transfer of a phosphate group between ATP and creatine, producing ADP and phosphocreatine [13]. The creatine kinase/phosphocreatine shuttle functions both as a subcellular energy transport system and as a temporal energy buffer [14]. Like arginine kinase, creatine kinase plays a central role in cellular energy metabolism [15]. Creatine kinase has been associated with various pathological conditions, including cancer, Alzheimer’s disease and Pick’s disease, muscular dystrophies, and myocardial infarction [16].

Typically, phosphagen kinases are composed of 300–400 amino acids and have a molecular weight of approximately 45 kDa [17]. They share a number of conserved sequence motifs that define the family [3]. Most arginine kinases exist as monomeric proteins, although dimeric forms have also been reported. In contrast, creatine kinases consistently function as obligate dimers. Additionally, duplicated forms of phosphagen kinases have been observed, likely arising from gene duplication events [18]. Structurally, phosphagen kinases are generally composed of an N-terminal α-helical bundle domain and a larger C-terminal domain [19]. The C-terminal domain is primarily responsible for nucleotide binding, and upon formation of a complex with a transition state analog, it undergoes an inward conformational shift that facilitates catalysis [15].

In this review, kinase protein structures are organized chronologically according to their date of publication. For each study, the most distinctive structural insight or novel finding is highlighted. Due to insufficient electron density, structurally incomplete data—despite their potential biological significance—were, regrettably, excluded in order to preserve the reliability of this review. Because of the structural similarity of homologs, overlapping findings from each study were not listed repeatedly. As a result, not all redundant discoveries or findings less directly related to structural characterization have been included.

## 2. Results

This study systematically analyzes the structural features of arginine kinase in its ligand-free and ligand-bound states. These conformations are commonly referred to as “open” and “closed” forms, respectively, due to significant rearrangements in domain positioning and loop dynamics. Notably, several structures of transition state analog complexes have since been reported, further validating this two-state model. The observed structural transitions not only illuminate the catalytic mechanism of arginine kinase but also provide a framework for understanding monomer-dimer interactions, allosteric regulation, and substrate-induced conformational changes across diverse species.

In this section, our primary goal is to present a species-by-species description of representative arginine kinase structures, allowing readers to clearly appreciate the unique characteristics of each enzyme from different organisms. To maintain clarity, each subsection was intentionally written as a self-contained narrative. Consequently, the order of presentation follows the chronological sequence in which these structures were determined.

### 2.1. The Crystal Structure of Chicken Cytosolic Brain-Type Creatine Kinase

**Figure 1:**
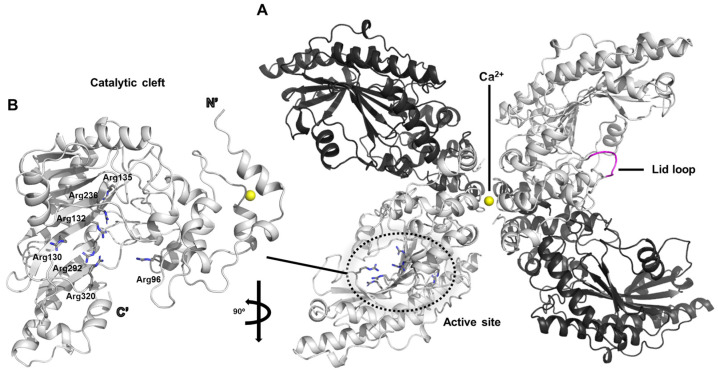
Overall structural representation of chicken brain-type creatine kinase. (**A**) The basic asymmetric unit comprising a tetrameric assembly is shown. Calcium ions involved in crystal stabilization are represented as yellow spheres. The flexible loop proposed to function as a lid regulating access to the active site is highlighted in purple. (**B**) Enlarged view of the catalytic cleft. Key arginine residues that contribute to substrate binding within the active site are labeled. The structure is rotated 90° relative to panel (**A**) to provide an alternative viewing angle of the active-site architecture.

The dimeric architecture of chicken creatine kinase adopts an elongated and curved conformation that is reminiscent of a banana-like shape (PDB ID: 1qh4) (Figure 1A) [20]. Within the crystallographic asymmetric unit, two such dimers are observed in parallel alignment, with calcium ion positioned along their central longitudinal axis. This calcium ion appears to play a pivotal role in mediating intermolecular interactions necessary for crystal packing and stabilization, thereby facilitating successful crystallization of the enzyme.

The active site of creatine kinase contains a dense cluster of arginine residues that are highly conserved across species. In particular, residues Arg96, Arg130, Arg132, Arg135, Arg236, Arg292, and Arg320 are consistently observed within the catalytic cleft (Figure 1B). Among these, Arg130, Arg132, Arg236, Arg292, and Arg320 are predicted to engage in specific electrostatic and hydrogen-bonding interactions with the phosphate groups of the nucleotide substrate, likely contributing to the stabilization of the transition state during phosphoryl transfer.

In the current crystal structure, the polypeptide segment encompassing residues 66 through 70 displays considerable conformational variability, as evidenced by markedly elevated average B-factors in this region. This flexible segment corresponds to a surface-exposed loop that is thought to act as a mobile “lid” over the active site, transiently closing over the substrate to shield the reaction center from bulk solvent (Figure 1A). This conformational plasticity is presumed to be functionally significant, allowing the enzyme to undergo necessary structural rearrangements that accommodate substrate binding and catalysis. Similar flexible loop mechanisms have been implicated in other members of the phosphagen kinase family, supporting the idea that dynamic gating of the active site is a conserved feature contributing to enzymatic efficiency [21].

In chicken cytosolic brain-type creatine kinase, the phosphoryl transfer reaction is achieved through the cooperative action of several conserved residues that precisely organize the substrates in the active site. The cluster of arginine residues (Arg130, Arg132, Arg236, Arg292, and Arg320) forms a strongly electropositive environment, anchoring the negatively charged phosphate groups of ATP or ADP by electrostatic attraction and hydrogen bonding. This arrangement stabilizes the transition state and properly orients the γ-phosphate for in-line transfer. Nearby residues, such as Arg96 and Arg135, contribute additional stabilizing contacts to the nucleotide and help guide the substrate into an optimal geometry. The flexible surface loop (residues 66–70) acts as a dynamic lid that closes over the bound substrates, shielding the active site from solvent and thereby reducing energy loss during the reaction. Together, these residues act in a concerted manner to position the reactants, stabilize the high-energy intermediate, and allow efficient phosphoryl transfer, a mechanism that appears to be conserved across the phosphagen kinase family.

### 2.2. The Structure of Torpedo Californica Creatine Kinase

**Figure 2:**
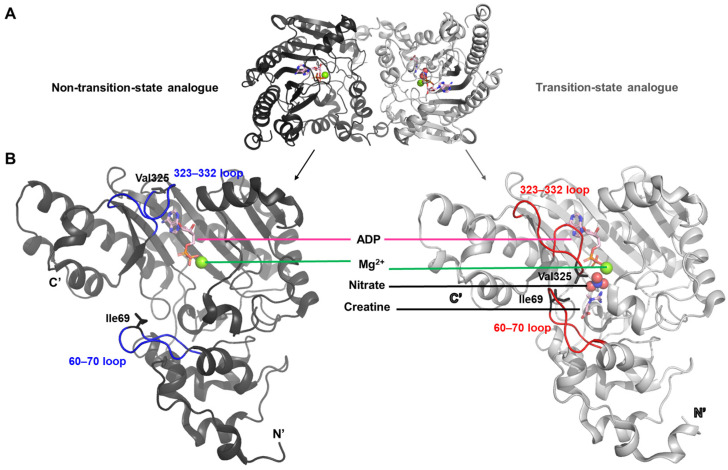
Overall structure of Torpedo californica creatine kinase and ligand-induced conformational changes. (**A**) Ribbon diagram showing the complete dimeric conformation. The left subunit represents the non–transition-state form, lacking creatine and nitrate, while the right subunit depicts the transition-state form bound to creatine, nitrate, Mg^2+^, and ADP. (**B**) Close-up comparison of the non–transition-state (black, **left**) and transition-state (gray, **right**) conformations. Two flexible loops that regulate the opening and closing of the active-site cleft are highlighted in blue and red, respectively. The transition-state structure illustrates how ligand binding induces an inward motion of these loops, resembling a grasping motion that encloses the active site.

This study presents the first X-ray crystal structure of creatine kinase in complex with a transition-state analogue [22]. Co-crystallization of the enzyme with ADP-Mg^2+^, nitrate, and creatine yielded a homodimer in which one monomer was fully occupied by all three ligands, while the other monomer contained only ADP and Mg^2+^ (PDB ID: 1vrp). The structure containing the complete set of ligands is referred to as the transition-state analogue complex (Figure 2A).

The transition between these two conformations reflects a structural rearrangement primarily driven by the movement of two flexible loop regions, spanning residues 60–70 and 323–332, respectively (Figure 2B). In the transition-state analogue complex, hydrophobic residues Ile69 and Val325—each located within one of the loops—are oriented inward toward the bound ligands. This arrangement appears to create a specificity pocket that enhances the binding affinity for ligands such as creatine or arginine.

The conformational transitions observed in *T. californica* creatine kinase are largely driven by ligand-induced interactions within the active site [22]. Binding of creatine and nitrate, in addition to ADP-Mg^2+^, introduces a network of stabilizing contacts that pull the two flexible loops (residues 60–70 and 323–332) inward. In particular, hydrophobic residues Ile69 and Val325 reorient toward the ligands, creating a complementary pocket that locks the substrates in place. This inward motion reduces solvent exposure and aligns catalytic residues for efficient phosphoryl transfer, effectively transforming the enzyme from an open to a closed state. The asymmetric arrangement of the dimer—one subunit fully occupied and closed, the other partially occupied and open—captures the sequential nature of this process, where ligand binding triggers a cascade of structural adjustments. These observations suggest that the driving forces behind the transition are primarily a combination of specific ligand–protein interactions, hydrophobic packing within the loops, and the entropic gain from reducing solvent-accessible surface in the active-site cleft.

The structure of this complex provides the first detailed view of the creatine-binding site in creatine kinase. The observed ligand-induced conformational shifts of the two flexible loops support the hypothesis that these elements undergo dynamic movement during substrate binding and catalysis.

### 2.3. Structural Studies of Human Brain-Type Creatine Kinase

**Figure 3:**
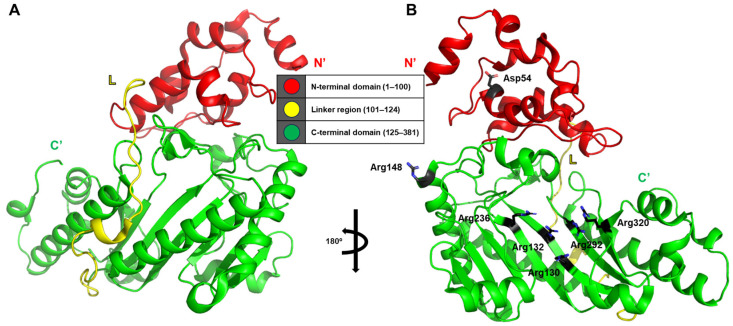
Structural features of human brain-type creatine kinase. (**A**) The overall structure of human brain-type creatine kinase consists of a small N-terminal domain (red), a linker region (yellow), and a larger C-terminal domain (green). These domains are indexed in the central schematic table using a traffic light–like color code. (**B**) Although not explicitly shown in this image, residues Asp54 and Arg148, which contribute to dimerization, as well as several conserved arginine residues that form the positively charged phosphate-binding pocket, are indicated in the full structural model.

In this study, the structure of human brain-type creatine kinase was elucidated using X-ray crystallography, with a detailed structural analysis achieved through domain-based partitioning of the molecule (PDB ID: 3dre) [13]. The crystallographic model of human brain-type creatine kinase reveals a helical N-terminal domain comprising residues 1–100 and a larger C-terminal domain adopting an α/β-fold architecture spanning residues 125–381. These two domains are connected by an extended linker region composed of residues 101–124 (Figure 3A).

Notably, the investigation provides a remarkable level of residue-specific insight into the structural interactions within and between monomers. One particularly meaningful observation is the interaction between Asp54 and Arg148 within a single monomer, which appears to contribute to the stabilization of the dimer interface through the formation of additional salt bridges or hydrogen bonds. Furthermore, five conserved arginine residues—Arg130, Arg132, Arg236, Arg292, and Arg320—form a positively charged phosphate-binding pocket that facilitates precise alignment and stabilization of the phosphoryl transfer reaction (Figure 3B).

In human brain-type creatine kinase, the phosphoryl transfer process is enabled by a cooperative network of conserved residues that precisely organize the substrates within the catalytic cleft. The core of this mechanism lies in a cluster of positively charged arginine residues (Arg130, Arg132, Arg236, Arg292, and Arg320), which form a phosphate-binding pocket that holds ATP or ADP in an orientation favorable for in-line transfer of the γ-phosphate. These residues form electrostatic and hydrogen-bonding interactions with the phosphate groups, stabilizing the transition state during catalysis. Additional structural support comes from the salt-bridge interaction between Asp54 and Arg148, which helps to stabilize the dimer interface and maintain the geometry of the active site. Once the substrates are positioned, the flexible regions surrounding the active site are thought to close in, reducing solvent exposure and creating a microenvironment optimized for efficient phosphoryl transfer. This cooperative interaction of charged residues, structural stabilization by interdomain contacts, and dynamic conformational closure illustrates how the enzyme achieves both precision and efficiency during catalysis, a mechanism conserved across the phosphagen kinase family.

These findings represent a significant contribution to our understanding of ligand-induced conformational changes, not only in human brain-type creatine kinase but also across homologous phosphagen kinases, offering structural context for conserved binding mechanisms observed in this enzyme family.

### 2.4. Arginine Kinase from the Sea Cucumber Stichopus Japonicus

**Figure 4:**
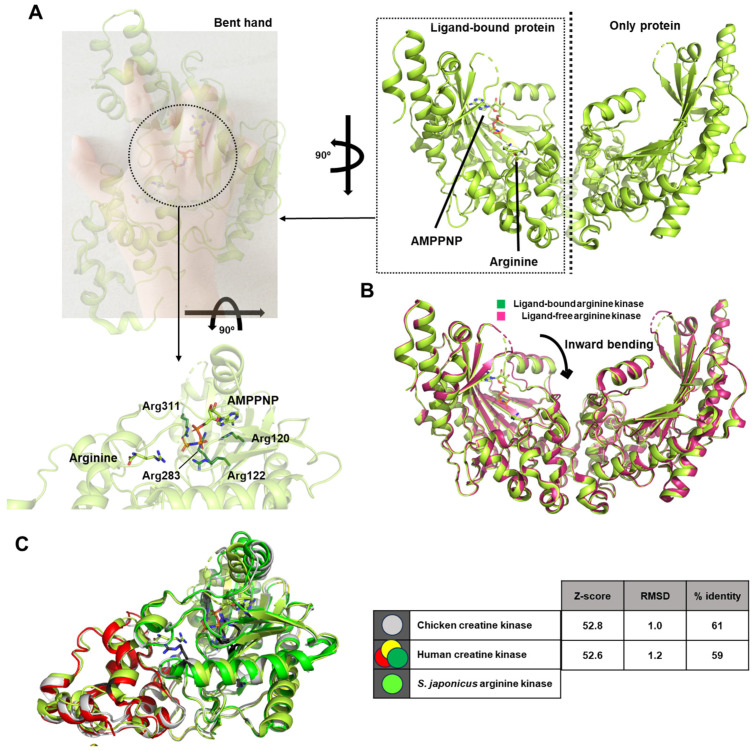
Representative summary of the structural findings on arginine kinase from the sea cucumber *Stichopus japonicus*. (**A**) Refer first to the overall homodimer shown on the right. Only the monomer on the left side is bound to ligands such as AMPPNP and arginine. A 90° rotated view of this ligand-bound monomer is shown on the left. The structure resembles a bent hand. The enlarged image below highlights the dotted circle from the left panel, where arginine residues forming the active site around the ligands are indicated in stick representation. (**B**) Superposition of the ligand-bound and ligand-free forms of arginine kinase. Although the difference is not dramatic, the authors of the study refer to this subtle shift as “inward bending,” a term retained here. (**C**) A figure and table comparing the overall structures and active sites of the enzymes described previously in Section 2.1 and Section 2.3. The color scheme used here follows the same colors as in each respective section. The statistical data are referenced to *S. japonicus* arginine kinase, which is the focus of the current Section 2.4.

In this study, the structure of arginine kinase, a member of the phosphagen kinase family, was resolved at a resolution of 1.75 Å, and the ternary complex structure with AMPPNP and arginine was also determined (PDB ID: 3ju5 and 3ju6) [23]. The resolved structure exists as two distinct dimers, with only one protein monomer bound to both AMPPNP and arginine. The authors described the three-dimensional fold of the monomer as resembling a bent hand, with the ligand-binding pocket located in the concave region of the palm (Figure 4A).

The biologically inert ATP analog AMPPNP is predicted to interact primarily through hydrogen bonding with several highly conserved, positively charged arginine residues. The authors characterized the associated structural rearrangement using the term “inward bending (Figure 4B)”. It is not considered a pronounced bending in my personal opinion; according to the authors, upon structural comparison, the ligand-bound arginine kinase was found to adopt a more widely open conformation than its ligand-free counterpart.

Given the spatial separation between the ligand-binding site and the region undergoing conformational change, the observed response is indicative of allosteric regulation. This implies that the conformational shift induced in one monomer upon ligand binding may weaken interprotomer interactions, ultimately leading to a significant reduction in the catalytic activity of the enzyme.

In the case of arginine kinase from the sea cucumber *S. japonicus*, the structural changes captured between the ligand-free and ligand-bound forms highlight an allosteric mechanism as the principal driver of the transition [23]. When arginine and the ATP analog AMPPNP occupy the active site, hydrogen bonding with conserved, positively charged residues stabilizes the substrates and triggers a subtle but coordinated rearrangement of the protein architecture. Rather than a pronounced closure, this effect has been described as a gentle “inward bending,” where the monomer containing ligands slightly shifts toward the active site, while the opposite monomer remains unbound and more open. Interestingly, this conformational change occurs despite the ligand-binding pocket being spatially distant from the regions that move, suggesting that local interactions propagate through the protein framework to influence interprotomer contacts. As a result, ligand-induced strain appears to reduce the strength of dimer interactions, a phenomenon that may modulate enzymatic turnover. These findings demonstrate that substrate binding initiates an allosteric cascade of structural adjustments, providing a mechanistic explanation for the observed differences in activity between the two monomers of the dimer.

In this study, the steady-state kinetic parameters determined for the enzyme indicated that the Michaelis–Menten constant (*K_m_*) for ATP was 6.1 ± 1.9 mM. This relatively high *K_m_* value suggests a moderate affinity of the enzyme toward ATP [23].

### 2.5. Crystal Structure of Shrimp Arginine Kinase from Litopenaeus Vannamei

In the 2010s, the structural elucidation of arginine kinase advanced significantly, shedding light on new biological roles beyond its classical function in energy metabolism (PDB ID: 4bhl) [24]. In particular, the arginine kinase from the Pacific white shrimp has been implicated in the organism’s immune responses to viral infections and to pattern recognition molecules derived from bacteria and fungi, such as peptidoglycan and laminarin [25]. These findings suggest a possible link between the innate immune system and the bioenergetic machinery in crustaceans [26].

**Figure 5:**
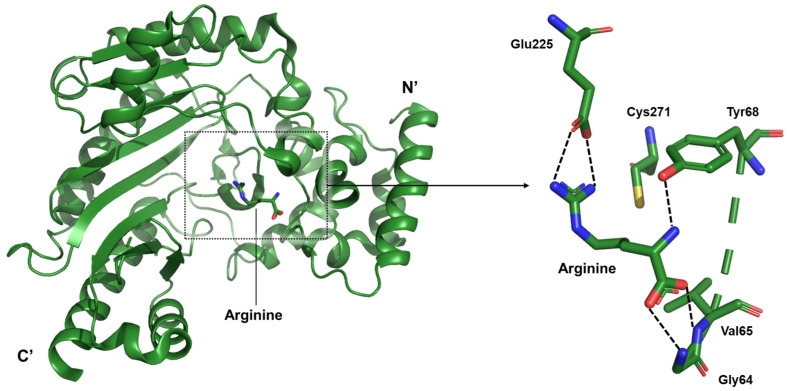
Structure of the Pacific white shrimp arginine kinase (green) and residue-specific interactions surrounding the bound arginine. Since no major conformational changes were observed upon arginine binding, comparison with the apo form is omitted. The dotted square in the left panel is magnified on the right, showing detailed residue interactions. The hydrogen bonding network described in the text is indicated.

The present study focuses on the arginine-bound state within the catalytic mechanism of arginine kinase. Structural analyses revealed that the guanidinium group of arginine engages in a strong ionic interaction with Glu225, a residue known to participate in acid–base catalysis during the phosphoryl transfer reaction. Glu225 forms hydrogen bonds with the terminal nitrogen atoms of the guanidinium moiety. On the zwitterionic end of the arginine molecule, the backbone amide nitrogens of Gly64 and Val65, along with the hydroxyl group of Tyr68, coordinate the carboxylate group, thereby stabilizing the substrate (Figure 5).

In the arginine kinase of the Pacific white shrimp *L. vannamei*, the structural basis for substrate recognition highlights how localized interactions guide and stabilize the phosphoryl transfer reaction [24]. This enzyme shows minimal global conformational change upon arginine binding, suggesting that the active site is already pre-organized to accept the ligand. Within the catalytic cleft, the guanidinium group of arginine forms strong ionic and hydrogen-bond interactions with Glu225, a residue that plays a central role in acid–base catalysis. Simultaneously, the carboxylate end of the substrate is anchored by a hydrogen-bonding network involving the backbone amides of Gly64 and Val65, together with the hydroxyl group of Tyr68. These interactions ensure proper alignment of the arginine molecule, positioning its reactive groups for the subsequent phosphoryl transfer. The absence of major loop closures or domain motions emphasizes a binding mechanism driven by precise residue positioning rather than large conformational shifts. This arrangement allows the enzyme to maintain catalytic readiness, supporting rapid turnover during periods of high energy demand.

### 2.6. Taurocyamine Kinase from the Human Parasite Schistosoma Mansoni

**Figure 6:**
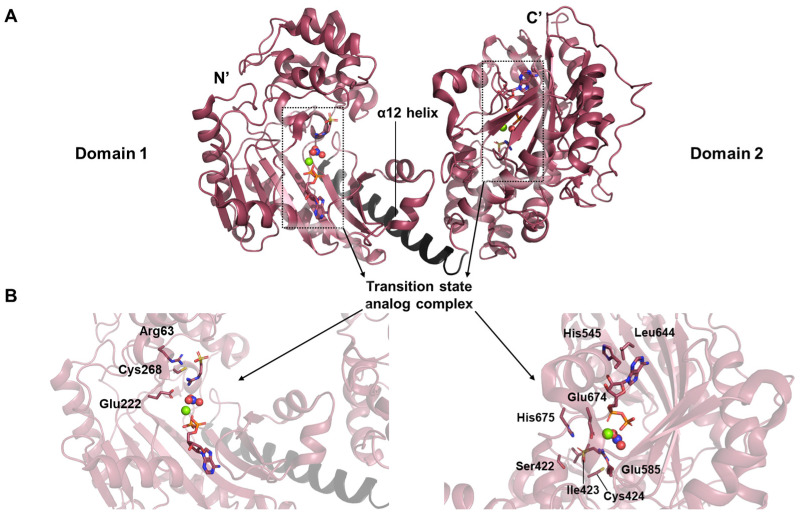
Structural analysis of taurocyamine kinase derived from *S. mansoni*. (**A**) The depicted image represents a single monomer, in which two distinct kinase domains are connected via the α12 helix. Each domain harbors a transition state analog complex within its active site. (**B**) Residue-specific interaction networks surrounding the transition state analog complexes in D1 and D2 are shown. The interactions described in the main text are illustrated. Although the figure presents distinct sets of interactions on the left and right corresponding to D1 and D2, it should be noted that these interactions are symmetrically present in both domains.

Taurocyamine kinase, a member of the phosphagen kinase family, catalyzes the reversible phosphoryl group transfer between ATP and taurocyamine in a magnesium-dependent manner [17]. Uniquely derived through gene duplication, this enzyme exists as a monomer in solution (PDB ID: 4woe). Structural investigation revealed an architecture distinct from previously characterized phosphagen kinases, comprising two continuous lobes—Domain 1 (D1) and Domain 2 (D2)—arranged in tandem (Figure 6A). Each domain corresponds to a fully functional kinase module, connected by an interface mediated through α12 helix.

The crystal structure further highlighted a tightly coordinated interaction network among the components of the transition state analog (Figure 6B). The transition state analog complex consists of substrate, nitrate, Mg^2+^, and ADP. Thanks to the high resolution of 1.9 Å, the interaction network around arginine in the D1 active site could be clearly resolved. The guanidinium group of arginine is oriented toward Glu222, with its inner nitrogen contacting the invariant Cys268. Meanwhile, the backbone nitrogen of the ligand lies adjacent to Arg63, contributing to the specificity and stabilization of substrate binding. In D2, His545 forms a hydrogen bond with the ribose moiety of the nucleotide, while Leu644 engages in stacking interaction with the adenine ring. Additionally, Glu674 plays a direct role in ligand binding, working alongside Glu585 to stabilize the substrate-binding pocket through hydrogen bonding. Other residues including His675, Ile423, Cys424, and Ser422 also participate in this stabilizing hydrogen bond network.

Within the transition-state analog complex, the binding of ADP was measured with a dissociation constant (*K_D_*) in the micromolar range, indicating a stable but non-covalent interaction that is characteristic of the closed, transition-state analog-stabilized conformation [17].

### 2.7. Structural Characterization of an Arginine Kinase from the Spider Polybetes Pythagoricus

In spiders, arginine kinase plays a dual biological role: it acts as a potent allergen and as part of the venom apparatus used to subdue prey, as demonstrated in both insects and spiders [27,28]. It is also essential for sustaining rapid predatory movement, providing immediate energy during muscular activity [29]. Due to its central role in invertebrate energy metabolism and its absence in vertebrates, arginine kinase has been proposed as a target for insecticides that disrupt energy regulation in pest species [30,31].

**Figure 7:**
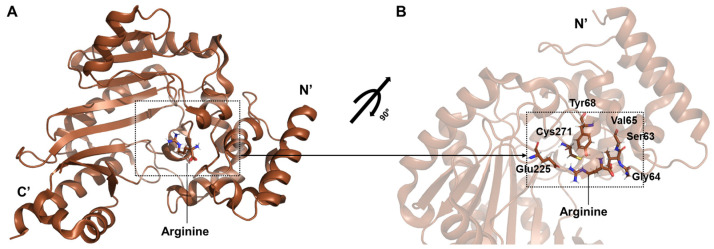
Structure of the spider arginine kinase (brown). (**A**) Overall structure of spider arginine kinase. (**B**) The bound arginine is surrounded by residue-specific interactions within the active site. These interactions are highly similar to those reported in shrimp arginine kinase studies. The dotted square in the left panel highlights the arginine-binding region. This region is enlarged in the right panel, where the interacting residues are labeled and illustrated in detail.

Two structural forms of spider arginine kinase obtained from muscle tissue were captured in the open conformation: an apo form and a binary complex with arginine (PDB ID: 5u92) (Figure 7A) [32]. The binary structure, in which only arginine is bound, reveals interactions consistent with other phosphagen kinases such as shrimp arginine kinase. Stabilizing hydrogen bonds are formed between arginine and residues Tyr68, Gly64, Val65, and Gly66, while Cys271 engages the guanidinium group to support catalysis. Glu225 contributes to proper substrate alignment for phosphoryl transfer (Figure 7B). These findings enhance the understanding of the conserved catalytic mechanism of arginine kinase and highlight its significance in arachnid bioenergetics and potential pharmacological exploitation.

A steady-state enzymatic kinetic analysis was carried out for *P. pythagoricus* arginine kinase using L-arginine as the variable substrate. The results showed a *K_m_* value of 1.7 mM, a *V_max_* of 27.8 μmol·min^−1^, and a turnover number (*k_cat_*) of 75 s^−1^, demonstrating that the enzyme functions as an efficient phosphotransferase in its physiological context [32].

### 2.8. Structure of McsB, a Protein Kinase from Geobacillus Stearothermophilus

The protein characterized in this study is McsB, an arginine-specific protein kinase [33]. In Gram-positive bacteria such as *G. stearothermophilus*, arginine phosphorylation plays a crucial role in maintaining protein homeostasis by regulating transcription factors and tagging misfolded proteins for degradation [34]. This structure reveals several novel and noteworthy architectural features.

**Figure 8:**
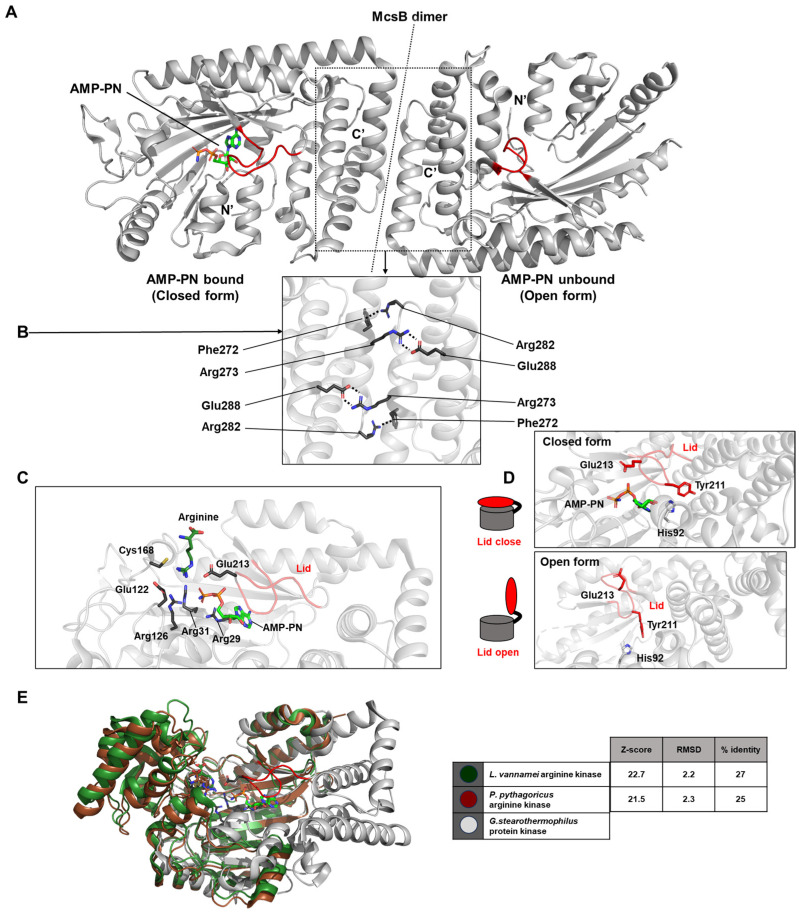
Structural overview of McsB. (**A**) The McsB dimer. The left subunit, bound to AMP-PN (closed form), and the right unbound subunit (open form) exhibit an asymmetric configuration. The lid loop that regulates access to the active site is highlighted in red. (**B**) The dimer interface. Key interacting residues contributing to dimerization are indicated. (**C**) The McsB active site with arginine modeled in. Arginine binding enhances the efficiency of the active site, thereby increasing the enzymatic activity. Key interacting residues and the lid loop are annotated. (**D**) Schematic illustration of lid loop opening and closing at the active site. Residues involved in lid motion and gating are labeled. (**E**) A figure and table comparing the overall structures and ligand-interaction networks of the enzymes described previously in Section 2.5 and Section 2.7. The color scheme used here follows the same colors as in each respective section. The statistical data are referenced to G. stearothermophilus protein kinase, which is the focus of the current Section 2.8.

McsB contains a domain resembling that of phosphagen kinases (PDB ID: 6fh2 and 6fh3). Structurally, McsB forms a flattened, “domino tile”-shaped dimer, stabilized by polar interactions such as a salt bridge between Arg273 and Glu288, and a cation–π interaction between Phe272 and Arg282 (Figure 8A,B). The active sites of both monomers comprising the dimer face the same surface. Each active site is coordinated by key residues including Arg29, Arg31, Arg126, Glu122, Glu213, and Cys168 (Figure 8C).

The conformation of the lid loop that covers AMP-PN determines whether the structure is in an open or closed state. The precise positioning of Glu213, which is regulated by interactions with Tyr211 and His92, is essential for the nucleotide-dependent opening and closing of the lid loop. When AMP-PN is bound to one domain, the lid is closed; in the unliganded domain, the lid remains open—resulting in an overall asymmetric conformation (Figure 8D). This structural asymmetry supports a mechanistic explanation for negative cooperativity in McsB, wherein substrate binding to one protomer enhances kinase activity via allosteric communication within the dimer.

In the case of McsB, both ATP and ADP displayed relatively weak binding affinities with dissociation constants of 262 ± 27 μM and 208 ± 60 μM, respectively. Additionally, the affinity of McsB for free phosphoarginine in solution was comparatively low, with a *K_D_* value of 850 ± 400 μM, consistent with the transient nature of pArg recognition [33].

### 2.9. The Arginine Kinase from the Tick Rhipicephalus Sanguineus

This study presents the high-resolution (1.53 Å) crystal structure of arginine kinase from the brown dog tick, *R. sanguineus*, in its substrate- and ligand-free open conformation [35]. The structure corresponds to the apo form, which does not form a complex with substrates or transition state analogues and represents the catalytically inactive, open state (PDB ID 7re6).

**Figure 9:**
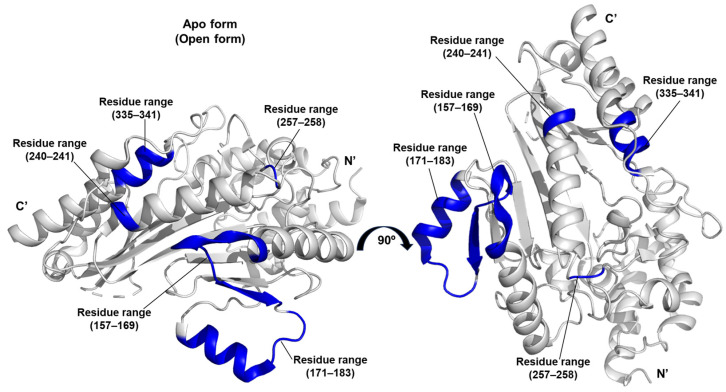
Predicted epitopes of arginine kinase from *R. sanguineus*. Residue ranges with antigenic potential, as predicted by the DiscoTope algorithm, are highlighted in blue.

A distinctive contribution of this work lies in the application of the DiscoTope algorithm to predict discontinuous epitopes and assess the antigenic potential of arginine kinase [36]. Based on structural data, the algorithm identified species-specific epitopes located within the residue ranges 157–169, 171–183, 240–241, 257–258, and 335–341. These predicted antigenic sites may serve as biomarkers of host exposure to arginine kinase (Figure 9).

The enzyme from *R. sanguineus* exhibited particularly high catalytic efficiency, as reflected by *k_cat_*/*K_m_^Arg^* = 523 and *k_cat_*/*K_m_^ATP^* = 458. These catalytic conversion constants underscore its effectiveness in phosphagen metabolism and highlight its prominent role in maintaining the energy homeostasis of this *R. sanguineus* [35].

### 2.10. Crystal Structure of Bacillus Subtilis McsB Kinase Domain Complexed with McsA

**Figure 10:**
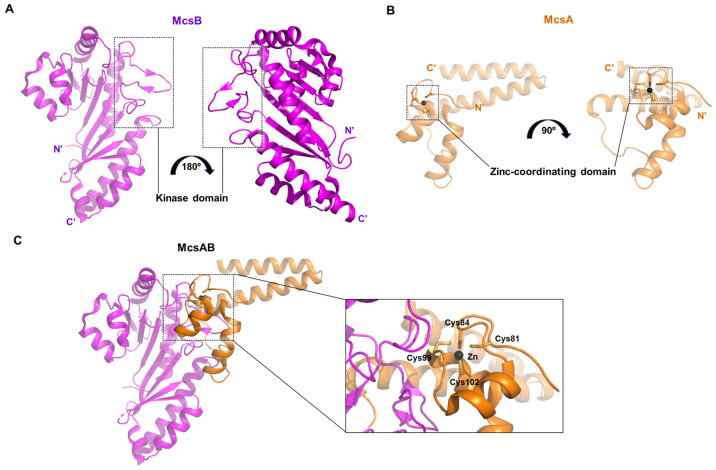
Protein structure information of McsB, McsA, and the McsAB complex from *B. subtilis*. (**A**) Structure of McsB and its 180° rotated view (purple). The kinase domain responsible for binding to McsA is indicated. (**B**) Structure of McsA and its 90° rotated view (orange). The zinc coordination domain involved in McsB interaction is highlighted. (**C**) Structure of the McsAB complex. The binding interface between McsA and McsB is enlarged and illustrated with the cysteine residues of McsA.

In this study, the crystal structure was elucidated not only for the protein arginine kinase McsB, but also for its activator McsA in complex with McsB (PDB ID: 8wtc) [34]. McsB, consisting of 257 amino acids (AA) due to partial proteolysis by chymotrypsin, is shorter than other kinases described earlier (Figure 10A). McsA is a small protein of approximately 100 AA, in which a zinc ion is coordinated by four cysteine residues (Cys81, Cys84, Cys99, and Cys102) (Figure 10B). This protein interacts with the kinase domain of McsB through its second zinc-coordinating domain and a subsequent loop region (Figure 10C). The association of McsA not only enhances the catalytic activity of McsB, but also prevents its self-oligomerization and promotes a stoichiometric binding ratio with its substrate [34].

## 3. Conclusions

Arginine kinase is indispensable in invertebrate physiology, particularly in tissues with high energy demands. The enzyme’s absence in vertebrates makes it an attractive target for developing species-specific inhibitors, particularly against pests and parasites. Structural insights—especially from transition state analog complexes—have revealed conserved mechanisms of conformational gating and phosphate transfer that are amenable to small-molecule targeting. Notably, the allergenic potential of arginine kinase in crustaceans opens further avenues in biomedical research, including diagnostics and therapeutics for food allergy control. Species-specific epitopes predicted by algorithms like DiscoTope may serve as biomarkers for exposure or as immunotherapeutic targets.

Predicted antigenic epitopes mapped onto the structure of arginine kinase provide a basis for structure-guided allergy control. These epitope maps can guide the development of diagnostic tools using epitope-specific antibodies and inform the design of modified proteins or synthetic peptides that reduce immunoglobulin binding. Such engineered hypoallergenic variants may serve as candidates for desensitization vaccines. By focusing on conserved surface-exposed epitopes, these structure-based approaches offer a rational path toward improved diagnosis and therapeutic strategies for allergies caused by arginine kinase in related species [35,37,38].

Structural studies of arginine kinase provide a foundation for exploring structure–activity relationships in inhibitor design. Detailed knowledge of substrate-binding pockets and conserved catalytic residues enables small molecules to be tailored for high-affinity binding, potentially blocking phosphoryl transfer. Comparative structural analyses across species suggest that conserved regions could allow broad-spectrum inhibitors, while subtle species-specific differences open the door for selective targeting. These insights imply that rationally designed inhibitors may not only disrupt energy metabolism in pests but could also be repurposed for therapeutic interventions in allergen-related pathways where arginine kinase plays a key role [34,39].

Although much progress has been made, key questions about arginine kinase remain. How the open–closed structural transitions are regulated in cells, particularly in dimeric and polydomain enzymes, is still unclear and may benefit from time-resolved crystallography or cryo-electron microscopy. Another important direction is defining species-specific structural differences that influence substrate binding, catalysis, and allergenicity, which could guide selective inhibitor or desensitization strategies. Understanding how these enzymes are integrated into broader energy homeostasis and regulated by partners or modifications also remains an open challenge [40,41].

Given the essential role of arginine kinase in energy metabolism, interfering with its function could impair physiological performance in arthropods such as ticks and shrimp, which are vectors or allergens. Future studies should explore the structural consequences of active-site mutations and the effect of small-molecule inhibitors on enzymatic activity. Additionally, integrating structural data with in vivo functional assays will be key to validating its druggability. Ultimately, these efforts could lead to the development of next-generation antiparasitic strategies and contribute to managing crustacean-related allergic reactions in humans.

## Abbreviations

The following abbreviations are used in this manuscript:

PDB	Protein Data Bank
ATP	Adenosine Triphosphate
AA	Amino acids
